# Modelling the Neurobiology of ADHD Using Human iPSC Systems: A Multimodal Platform for Mechanistic Discovery

**DOI:** 10.3390/cells15100931

**Published:** 2026-05-19

**Authors:** Atefeh Namipashaki, Hanchen Yu, Mark A. Bellgrove, Ziarih Hawi

**Affiliations:** 1 Turner Institute for Brain and Mental Health, School of Psychological Sciences, Monash University, Melbourne, VIC 3800, Australia; 2 Department of Anatomy and Physiology, The University of Melbourne, Parkville, VIC 3010, Australia

**Keywords:** ADHD, human iPSCs, neurodevelopment, dopaminergic signalling, excitation/inhibition balance, disease modelling

## Abstract

Attention deficit hyperactivity disorder (ADHD) is a common and highly heterogeneous neurodevelopmental condition with complex biological underpinnings. Despite substantial progress in identifying genetic and neurobiological correlates, the cellular mechanisms linking genetic variation to functional brain alterations remain poorly understood. Human induced pluripotent stem cell (iPSC) technology provides a powerful platform to investigate these mechanisms by enabling the generation of patient-specific neural cell types and the direct interrogation of molecular, cellular, and network-level phenotypes. In this review, we summarise the current understanding of the neurobiological mechanisms underlying ADHD, including dopaminergic dysregulation, delayed neurodevelopmental maturation, and excitatory/inhibitory imbalance. We then discuss how iPSC-based models, combined with genome engineering and advanced functional assays, can be used to dissect gene-specific effects, study neural circuit development, and establish scalable platforms for therapeutic discovery. Finally, we outline key methodological considerations for designing robust iPSC-based models of ADHD. Together, these approaches provide new opportunities to bridge genetic risk with cellular function and accelerate the development of mechanistically informed therapeutic strategies.

## 1. Introduction

Attention deficit hyperactivity disorder (ADHD) is the most common neurodevelopmental condition, affecting approximately 5–6% of children and 2–3% of adults worldwide, with symptoms persisting into adulthood in at least 60% of adults [1]. It is characterised by persistent patterns of inattention and/or hyperactivity/impulsivity that interfere with academic, occupational, and social functioning [2]. ADHD imposes a substantial burden on individuals, families, and society, contributing to educational underachievement, occupational difficulties, increased risk-taking behaviours, accidental injuries, and elevated rates of psychiatric comorbidity [3]. The economic impact of ADHD is considerable, with global costs estimated in the hundreds of billions of dollars annually, reflecting healthcare utilisation, reduced productivity, and broader societal consequences [4].

Current pharmacological treatments for ADHD primarily target catecholaminergic neurotransmission. Stimulants, including methylphenidate and amphetamine derivatives, are recommended as first-line therapy and increase synaptic dopamine and norepinephrine to alleviate core symptoms [4]. Non-stimulant medications are used when stimulants are ineffective or poorly tolerated. Although current treatments can alleviate symptoms by enhancing monoaminergic transmission, they do not address the underlying neurodevelopmental alterations associated with ADHD [5]. Importantly, some individuals respond poorly or experience adverse effects, while symptom recurrence after treatment discontinuation and high psychiatric comorbidity further complicate clinical management [2,6,7]. These limitations highlight the need for therapies that move beyond symptom management toward targeting the underlying mechanisms of ADHD biology.

In this review, we summarise current knowledge of the neurobiological mechanisms underlying ADHD and highlight how induced pluripotent stem cell (iPSC) technology can be leveraged to investigate those related functional processes at the cellular and molecular resolutions. We discuss how iPSC-based models provide a platform to dissect gene-specific and circuit-level alterations, offering new opportunities to bridge genetic risk with functional phenotypes, informing future therapeutic approaches. Finally, we outline key methodological and conceptual considerations for establishing robust iPSC-based models of ADHD.

## 2. Converging Neurobiological Mechanisms in ADHD

**Figure 1:**
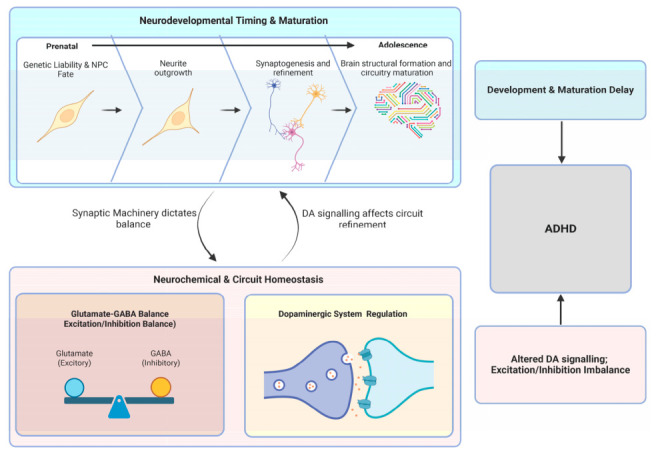
Proposed neurobiological mechanisms underlying ADHD. A conceptual framework illustrating how interacting neurodevelopmental and neurochemical processes may contribute to ADHD. During brain development, from the prenatal period through adolescence, genetic liability can influence neural progenitor cell fate and subsequent stages of neuronal maturation, including neurite outgrowth, synaptogenesis, and circuit formation. Disruptions in the timing or progression of these developmental processes may lead to delayed maturation of neural networks. In parallel, neurochemical and circuit homeostasis is regulated by balanced interactions between excitatory glutamatergic and inhibitory GABAergic signalling, as well as dopaminergic system regulation. Alterations in dopaminergic signalling and excitation/inhibition balance can disrupt synaptic machinery and neural circuit stability. Together, delayed neurodevelopmental maturation, altered neurotransmitter regulation and excitation/inhibition imbalance may converge to produce the cellular and circuit-level dysfunctions associated with ADHD. Created in BioRender. namipashaki, A. (2026) https://BioRender.com/tte0boq.

Multiple neurobiological mechanisms have been proposed to underlie ADHD, converging on alterations in catecholaminergic signalling, neurodevelopmental timing and maturation, and more recently, excitatory/inhibitory (E/I) balance within fronto-striatal circuitry [4,8]. A summary of the key neurobiological mechanisms is shown in Figure 1. While historically examined as independent hypotheses, emerging evidence suggests that these mechanisms are highly interrelated and may reflect different levels of dysfunction within shared neural systems.

### 2.1. Dopaminergic System Dysregulation

The dopaminergic hypothesis of ADHD was first articulated by Levy in the late 20th century and remains one of the most robustly supported neurobiological frameworks for the disorder [9]. Dysregulation of dopamine signalling is thought to play a central role in ADHD pathophysiology and has been linked to the core symptoms of inattention, impulsivity, and hyperactivity [10]. Several lines of evidence support this hypothesis. Pharmacological evidence including stimulant medications (methylphenidate and amphetamine), block dopamine and norepinephrine transporters and increase synaptic catecholamine levels, are the first-line treatment for ADHD and demonstrate strong clinical efficacy [11]. Their therapeutic effects provide indirect evidence for catecholaminergic dysregulation in ADHD. Further, variants in genes related to dopamine transmission, including the dopamine transporter (*DAT1*/*SLC6A3*) and dopamine receptors (e.g., *DRD4*), have been associated with ADHD risk [12,13]. Animal models further strengthen this hypothesis; *Dat1* knockout mice exhibit hyperactivity and impaired inhibitory control, phenotypes reminiscent of ADHD [14,15].

Accumulating evidence indicates that dopamine plays a central role in regulating neural circuits that support attention, cognitive control, and behavioural inhibition, processes that are commonly altered in ADHD [16,17]. Dopaminergic signalling is particularly important for coordinating activity within interconnected cortical and subcortical brain regions involved in executive function [18]. Through its actions on dopamine receptors, dopamine modulates neuronal excitability, synaptic transmission, and the balance of activity within these circuits, thereby influencing the prioritisation of relevant information and the suppression of inappropriate responses [19]. The disruption of dopaminergic signalling is therefore thought to alter the functional organisation of these neural networks, contributing to impairments in attention regulation, impulsivity, and behavioural control observed in ADHD [20].

### 2.2. Altered Neurodevelopmental Timing and Delayed Maturation

In addition to catecholaminergic dysregulation, ADHD also manifests as a disorder of altered neurodevelopmental timing, reflecting disruptions in the coordinated processes that guide brain maturation [21]. Neurodevelopment of the human brain is a highly coordinated process that begins prenatally and continues into early adulthood, involving sequential stages such as neural stem cell proliferation, neuronal migration, neurite outgrowth, synaptogenesis, circuit refinement, and large-scale network maturation. Disruptions to the timing or regulation of these processes are increasingly thought to contribute to the pathophysiology of ADHD [4].

Recent stem cell-based studies provide mechanistic insight into early developmental alterations at the cellular level. For example, reduced proliferation rates have been observed in ADHD-derived neural stem cells, suggesting slowed neurodevelopmental progression at the progenitor stage [22]. Such early alterations may propagate forward, influencing neuronal differentiation timing, neurite complexity, and synaptic maturation. At the macroscale, neuroimaging studies demonstrate delayed cortical maturation in ADHD. Peak cortical thickness, particularly in prefrontal regions, occurs several years later in children with ADHD compared to typically developing controls [23]. Large-scale ENIGMA consortium analyses further report altered cortical surface area and reduced subcortical volumes, especially within fronto-striatal circuits [24]. These findings support the “maturation delay hypothesis” suggesting that ADHD reflects altered timing of cortical thinning, synaptic pruning, and network specialisation rather than overt structural abnormalities alone [25,26].

### 2.3. Excitatory/Inhibitory (E/I) Imbalance

A growing body of literature implicates altered E/I balance as a core neurobiological mechanism in ADHD, involving both the GABAergic and glutaminergic dysfunction [27]. Magnetic resonance spectroscopy (MRS) studies report reduced GABA concentrations in key regions implicated in inhibitory control, including the motor cortex and somatosensory cortex [28], striatum in children with ADHD, correlating with impaired inhibitory control [28], and anterior cingulate cortex in adults with ADHD [29]. Consistent with these findings, reduced GABAergic signalling within the prefrontal cortex and anterior cingulate cortex has been associated with impaired response inhibition and increased impulsivity [30].

Genetic studies further support the involvement of inhibitory pathways, identifying associations between ADHD and variants in GABA-related genes, including polymorphisms in *GAD1*, which encodes glutamic acid decarboxylase, a key enzyme in GABA synthesis [31]. Additional studies also highlight genes regulating GABAergic interneuron development and synaptic function, reinforcing the link between inhibitory circuitry and ADHD [32].

In parallel, elevated glutamate or glutamate/glutamine (Glx) levels have been reported in the prefrontal cortex and striatum of individuals with ADHD [33,34], suggesting a relative shift toward increased excitatory signalling. Together with reduced GABAergic inhibition, this imbalance may lead to increased cortical excitability. Supporting this view, oscillatory and spectral EEG studies also report altered patterns of brain activity in ADHD, which may reflect disruptions in the balance between excitatory and inhibitory signalling within cortical circuits [8].

### 2.4. Integrative Perspective: Interdependence of Mechanisms

The proposed biological mechanisms of ADHD, including dopaminergic dysregulation, altered neurodevelopmental timing, and E/I imbalance, are not mutually exclusive mechanisms. Instead, they likely represent interacting levels of dysfunction across molecular, cellular, and circuit scales. Dopaminergic signalling interacts closely with neurodevelopmental processes and plays a key modulatory role in cortical and subcortical circuits by regulating both GABAergic interneuron activity and glutamatergic synaptic transmission, thereby influencing network excitability and information processing [16,35]. In addition, dopamine influences progenitor proliferation, neuronal differentiation and wiring during development [36,37]. Consequently, dopaminergic dysregulation may not only alter neurotransmission but also shape developmental trajectories.

In parallel, disruptions in E/I balance during early development can influence synaptic pruning, neuronal maturation, and circuit refinement, processes that are essential for the formation of stable neural networks [38,39]. Consistent with this view, delayed cortical maturation observed in ADHD may reflect disrupted activity-dependent stabilisation of neural circuits, a process tightly regulated by balanced excitatory and inhibitory signalling [17,23].

Within this framework, early alterations in neural progenitor proliferation, interneuron maturation, or synaptic development could shift the E/I balance, disrupt dopaminergic modulation of fronto-striatal circuits, and ultimately impair the emergence of stable executive networks. These cumulative disruptions may manifest clinically as persistent symptoms of inattention, impulsivity, and hyperactivity. Taken together, ADHD may be best conceptualised as a disorder of circuit maturation and network regulation, in which altered catecholaminergic signalling, delayed neurodevelopmental timing, and E/I imbalance converge on fronto-striatal systems [10,17,18]. Understanding how these mechanisms interact across developmental stages will be critical for designing mechanistically driven precision therapeutic strategies.

## 3. Application of iPSC Technology to Study the Biological Mechanisms of ADHD

iPSC technology provides a powerful and human-relevant platform to investigate the neurobiological mechanisms underlying ADHD. As a complex neurodevelopmental condition involving distributed cortical and subcortical circuitry, ADHD cannot be fully recapitulated in animal models, which lack the genetic architecture and higher-order network organisation of the human brain [40]. Moreover, access to living human neural tissue is extremely limited [41]. iPSC-based systems therefore offer a transformative approach by enabling the study of patient-specific neural cells in vitro while preserving the individual’s genetic background.

**Figure 2:**
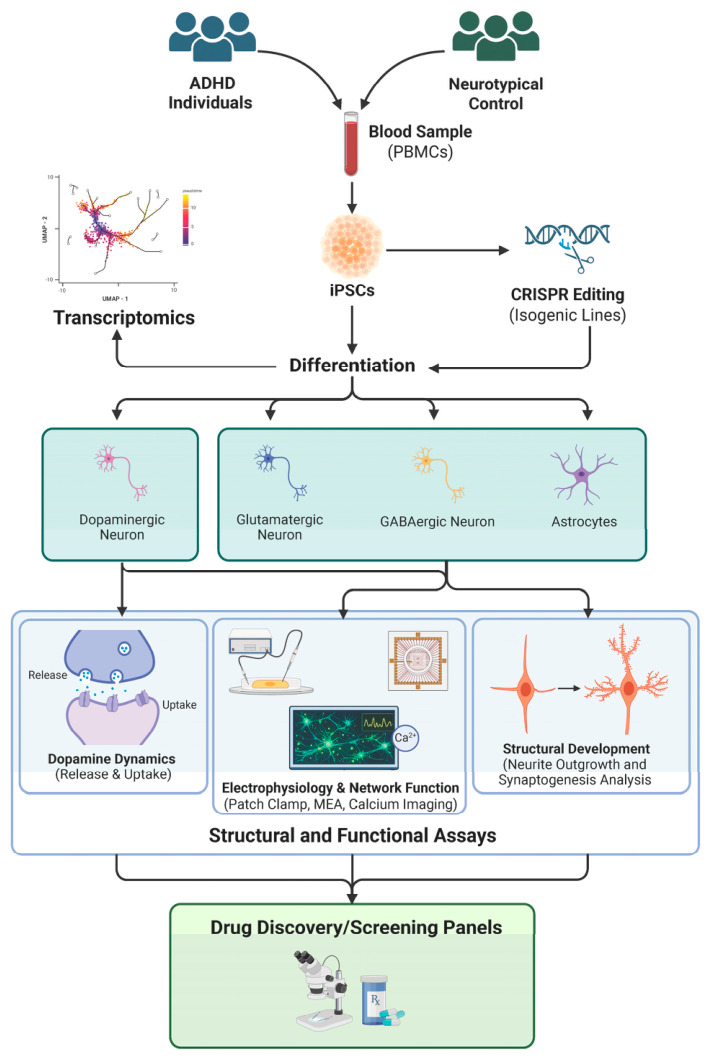
iPSC-based framework for investigating molecular and cellular mechanisms of ADHD and path to therapeutic discovery. Schematic overview of an iPSC platform to model ADHD biology. PBMCs obtained from individuals with ADHD and neurotypical controls are reprogrammed into iPSCs. Genome engineering approaches such as CRISPR/Cas9 can be applied to generate isogenic lines to interrogate gene-specific effects. iPSCs are subsequently differentiated into relevant neural cell types, including dopaminergic, glutamatergic, and GABAergic neurons, as well as astrocytes. These models enable the investigation of multiple disease-relevant phenotypes through integrated structural and functional assays, including neurite outgrowth and synaptogenesis analysis, electrophysiological and network activity measurements (patch clamp, multielectrode array, and calcium imaging), and dopamine release and uptake dynamics. In parallel, transcriptomic analyses can capture developmental trajectories and molecular signatures of ADHD-derived neurons. Together, these approaches provide a human-relevant experimental framework for studying neurodevelopmental mechanisms, facilitating the path for future therapeutic screening efforts. Created in BioRender. namipashaki, A. (2026) https://BioRender.com/tte0boq.

iPSC models capture patient-specific genetic liability and allow scalable, reproducible generation of quantitative cellular phenotypes suitable for mechanistic interrogation and therapeutic screening. Through controlled differentiation protocols, iPSCs can be directed into neural cell types implicated in ADHD, enabling direct measurement of structural, electrophysiological, synaptic, and neurotransmitter-related phenotypes [42]. A proposed workflow illustrating how iPSC platforms can recapitulate ADHD-related biological mechanisms in vitro is shown in Figure 2.

### 3.1. Experimental Design

When designing experiments for analysis, several key factors should be considered before determining the study design, experimental readouts, and downstream applications. Below is an overview of the workflow applicable in the development, interpretation, and translational relevance of the experiments.

#### 3.1.1. iPSC Modelling Approaches

iPSC lines can be generated from peripheral blood mononuclear cells (PBMCs) or fibroblasts obtained from individuals with ADHD and neurotypical controls. Two complementary experimental strategies can be employed. In a case–control cohort design, a larger cohort-based approach enables the identification of convergent cellular and functional phenotypes associated with ADHD across individuals. This strategy is particularly useful for capturing polygenic risk and population-level variability [43]. However, detecting subtle differences often requires sufficiently powered cohorts due to inter-individual variability [44].

The other approach is to generate isogenic CRISPR-based models. To reduce genetic background variability, CRISPR/Cas9 genome editing can be integrated with iPSC technology to introduce or correct ADHD-associated variants, knock out candidate genes, or model GWAS-identified loci [45]. Isogenic lines differ only at the specific genomic region of interest, enabling the precise attribution of observed phenotypes to defined genetic perturbations [46]. This approach is especially powerful for dissecting the functional impact of emerging ADHD risk genes identified through large-scale genome-wide association studies. Both of the cohort-based and isogenic strategies provide complementary insights into both polygenic architecture and gene-specific mechanisms.

#### 3.1.2. Neuronal Cell Types Relevant to ADHD

Once established, iPSC lines can be differentiated into neural cell populations relevant to ADHD pathophysiology. These include but are not limited to dopaminergic neurons, glutamatergic neurons, GABAergic neurons and region-specific cortical projection neurons [28,47,48,49,50,51]. Astrocytes and other glial regulators of network homeostasis can also be included [50]. These cell types encompass key excitatory and inhibitory components of cortical and striatal circuits, as well as non-neuronal modulators of synaptic and network function. Cultures can be maintained as monocultures to isolate cell-autonomous effects or as co-cultures to model excitatory/inhibitory interactions and circuit-level dynamics.

### 3.2. A Multimodal Framework to Study iPSC Derived Neurons: Readouts and Applications

iPSC-derived neurons can be subjected to a multimodal framework of structural, functional, and molecular assays to interrogate ADHD-relevant mechanisms.

#### 3.2.1. Neurotransmitter Signalling Assays

Dopaminergic neurons enable the direct investigation of dopamine biology, including dopamine release and uptake assays or transporter and receptor function assays. These tests have been facilitated by using genetically encoded dopamine sensors [52,53]. These experiments directly address the long-standing dopaminergic hypothesis of ADHD and allow mechanistic testing of synaptic dopamine regulation.

#### 3.2.2. Functional Maturation Analysis

Neuronal and structural maturation can be assessed through neurite outgrowth quantification during differentiation to assess dendritic and axonic complexity, and through high-resolution imaging of synaptogenesis and its marker’s expression [54,55]. Such structural assays are particularly relevant for testing hypotheses related to delayed neurodevelopmental timing and impaired synaptic maturation.

Further, functional maturation and network activity can be evaluated longitudinally using calcium imaging to assess calcium dynamics, whole-cell patch clamp electrophysiology for intrinsic membrane and synaptic properties, and multielectrode array recordings to capture emergent network synchrony and oscillatory dynamics [56,57,58]. These platforms allow for an investigation of both single-cell excitability and higher-order network organisation. Importantly, excitatory/inhibitory balance can be directly interrogated in co-culture systems combining glutamatergic and GABAergic neurons, enabling quantification of circuit-level dysregulation.

#### 3.2.3. Molecular Assays

Genome-wide transcriptional profiling across differentiation time points enables reconstruction of developmental trajectories and identification of altered maturation programs. Integrating temporal transcriptomics with functional readouts can reveal molecular pathways contributing to delayed neuronal maturation, altered synaptic development, or dysregulated neurotransmission. Once robust phenotypic assays are established, these systems can be adapted into high-throughput platforms for drug discovery. Phenotype-based screening in genetically defined iPSC models allows for the identification of compounds that rescue altered cellular or network phenotypes. This approach moves beyond symptomatic treatment toward mechanistically informed therapeutic strategies.

## 4. Key Considerations When Using iPSC-Derived Models of ADHD

While iPSC platforms offer unprecedented opportunities to investigate the biological basis of ADHD, careful study design and cohort selection are critical to ensure robust and interpretable findings. In particular, when implementing case–control study designs, adequate sample size is essential to account for inter-individual variability arising from heterogeneous genetic backgrounds. This consideration is especially important for ADHD, which has a highly polygenic genetic architecture, where risk is distributed across many common variants with small individual effects [59,60]. As a result, cellular phenotypes observed in patient-derived models may be subtle and influenced by cumulative genetic burden rather than single causal variants. Consequently, sufficiently powered cohorts are required to detect reproducible biological differences and to distinguish disease-relevant phenotypes from background genetic variability [44].

Although recruiting large cohorts is logistically challenging, international collaboration offers a practical solution. Establishing global consortia to pool iPSC lines can substantially increase statistical power, improve reproducibility, and enhance representation across diverse ethnic backgrounds [61,62]. Table 1 provides an overview of published ADHD patient-derived iPSC lines worldwide and their associated functional findings. While the total number of reported iPSC lines is listed as *n* = 73, an examination of the cell identifiers suggests that several studies have used overlapping cell lines, and others have reported multiple clones derived from the same individual as separate lines. Although the inclusion of multiple clones from a single donor is important for accounting for the inherent variability of iPSC models, the actual number of unique individuals represented across studies appears to be closer to *n* = 25.

Across published studies, ADHD-derived neuronal models have recurrently demonstrated alterations in proliferation and maturation rates, supporting the hypothesis of disrupted neurodevelopmental timing and neuronal maturation in ADHD [22,47,63]. In addition, individual studies have reported alterations in pathways associated with polyunsaturated fatty acid responses, excitation/inhibition balance, oxidative stress, and related neurobiological processes [63,73]. However, many of these findings are based on relatively small sample sizes, limiting statistical power and reproducibility across studies. Notably, only a single study to date has employed a 3D organoid-based approach, highlighting a major gap in the field [47]. Expanding the application of organoid and other advanced multicellular models may provide more physiologically relevant systems for investigating ADHD-associated neurodevelopmental mechanisms. Collectively, coordinated efforts to standardise differentiation protocols, phenotyping pipelines, and data-sharing practices across existing iPSC resources would substantially accelerate progress in the field and facilitate large-scale cross-validation studies.

The comprehensive clinical characterisation of donors is equally important. ADHD is clinically heterogeneous, encompassing inattentive, hyperactive/impulsive, and combined presentations, as well as dimensional variation in symptom severity [74,75]. Stratifying iPSC experiments according to symptom domains or cognitive endophenotypes may allow for a stronger alignment between observed cellular phenotypes and clinical features [76,77,78]. Such approaches move beyond binary case–control comparisons toward biologically informed subclassification.

Given the strong polygenic nature of ADHD, calculating polygenic risk scores (PRSs) for all donor lines including both ADHD and neurotypical controls can provide an additional layer of biological interpretation [79]. PRS may help explain variability in cellular phenotypes and enable correlation analyses between cumulative genetic risk and in vitro functional outcomes. In addition, genomic characterisation through whole-exome or whole-genome sequencing is valuable to identify rare variants, copy number variations, or de novo mutations that may influence neuronal phenotypes. Integrating common variant burden (PRS) with rare variant analysis allows for a more precise interpretation of experimental results [80].

Consideration of comorbidities and other confounding factors is also essential. ADHD frequently co-occurs with a range of psychiatric and medical conditions, including externalising and internalising disorders and other neurodevelopmental conditions such as autism spectrum disorder, as well as bipolar disorder, substance use disorders, and metabolic or sleep disturbances [81]. Failure to account for comorbid conditions may introduce confounding biological signals that obscure ADHD-specific mechanisms. Careful screening during sample recruitment and clear inclusion/exclusion criteria are therefore essential. When comorbidities are present, stratified analyses may help disentangle overlapping versus disorder-specific mechanisms.

For isogenic models, when integrating CRISPR/Cas9 genome editing to generate isogenic lines, rigorous clonal validation is essential. Experiments should be performed on single-cell-derived clones to minimise mosaicism and heterogeneity. Off-target effects must be assessed, and multiple independent clones should ideally be analysed to confirm reproducibility of phenotypes [46]. Established protocols that enable efficient yet homogeneous editing of iPSCs are particularly valuable in this context. High-quality genome engineering workflows reduce clonal variability and strengthen causal inference when modelling ADHD-associated variants or gene perturbations [82].

## 5. Limitation of Using iPSC-Based Models in ADHD Research

Despite their substantial potential, iPSC-based models also possess important limitations that must be carefully considered when interpreting findings in ADHD research. One of the major challenges is the reproducibility and consistency of reported phenotypes across studies. Considerable variability exists between iPSC lines due to differences in donor genetic background, reprogramming methods, clonal selection, passage number, and culture conditions [44,61]. In addition, neuronal differentiation protocols vary substantially between laboratories with respect to patterning strategies, maturation timelines, media composition, and cell-type specification, making direct comparison between studies difficult [83]. Such methodological heterogeneity may contribute to inconsistent or non-overlapping findings across ADHD iPSC studies and complicate interpretation of disease-relevant phenotypes.

Another important limitation is that iPSC-derived neurons frequently exhibit an immature developmental state resembling foetal or early postnatal neurons rather than fully mature adult neural phenotypes [84]. Although this feature may be advantageous for studying early neurodevelopmental processes, it can limit the modelling of later-stage neuronal maturation, long-range circuit integration, and age-dependent functional properties relevant to ADHD. In addition, most current iPSC systems cannot fully recapitulate the structural and cellular complexity of the human brain in vivo, including vascularization, microglial interactions, long-range connectivity, and dynamic environmental influences [85]. Even advanced three-dimensional organoid systems remain limited by incomplete regional organisation, variability between organoids, and restricted maturation [86].

Consequently, the findings obtained from in vitro iPSC models should be interpreted cautiously and ideally integrated with complementary approaches including animal models, neuroimaging studies, and human genetic analyses. Future progress in the field will likely depend on greater protocol standardisation, larger collaborative cohorts, improved neuronal maturation strategies, and the development of more physiologically complex multicellular systems capable of better modelling human neural circuitry.

## 6. Conclusions

In conclusion, ADHD arises from complex interactions between genetic risk, catecholaminergic regulation, neurodevelopmental timing, and neural circuit regulation. Human iPSC-based models provide a promising human-relevant platform to investigate these mechanisms by linking genetic variation to cellular and network-level phenotypes. By integrating patient-derived models with advanced functional assays and genome engineering approaches, iPSC technology offers new opportunities to uncover disease mechanisms and accelerate the development of mechanistically informed therapeutic strategies for ADHD.

## Figures and Tables

**Table 1:** Overview of published iPSC lines derived from individuals with ADHD (*n* = 73).

Cell Identifier	ADHD Type	No. of Lines	Clinical Notes	Donor Details		Neuro Differentiation & Functional Findings	Ref.
				Age	Sex		
MR001	ADHD-I	2	Methylphenidate responder; PRS z = −0.65	15	Male	Polyunsaturated fatty acids regulate Wnt activity in some ADHD-derived neural stem cells	[63]
MR010	ADHD-I	2	Methylphenidate responder; PRS z = 1.62	9	Male		
MR013	ADHD-C	2	Methylphenidate responder; PRS z = 0.29	16	Male		
MR014	ADHD-C	2	Methylphenidate responder; PRS z = 0.45	13	Male		
MR030	ADHD-C	2	Methylphenidate responder; PRS z = 1.13	9	Female		
NR002	ADHD-C	2	Methylphenidate non-responder; PRS z = 1.64	13	Male		
NR003	ADHD-C	2	Methylphenidate non-responder; PRS z = −0.02	14	Male		
MR012	ADHD	2	Methylphenidate responder	11	Male *	QC only	[64]
MR030	ADHD	2	Methylphenidate responder	9	Female *		
NR002	ADHD	2	Methylphenidate non-responder	13	Male *		
NR002	ADHD	2	Methylphenidate non-responder	14	Male *		
MR013	ADHD	1	Methylphenidate responder	16	Male *	QC only	[65]
MR023	ADHD	2	Methylphenidate responder	12	Male *		
UKWi002-A	ADHD	2	ADGRL3 rs1397547 wildtype (C/C)	27	Male *	Genotype-stratified lines (ADGRL3); QC only	[66]
UKWi005-A	ADHD	2	ADGRL3 rs1397547 wildtype (C/C)	46	Male *		
UKWi003-A	ADHD	2	ADGRL3 rs1397547 heterozygous (C/G risk)	39	Male *		
UKWi004-A	ADHD	2	ADGRL3 rs1397547 heterozygous (C/G risk)	39	Male *		
ADHD-(1,2,3)	ADHD	3	DSM-IV-TR ADHD	18	Male	ADHD-derived telencephalon organoids show thinner cortex layer and contain more neurons, and also show changes in proliferation and apoptosis	[47]
MR001	ADHD	2	Methylphenidate responder; PRS z = 0.13	15	Male	ADHD-derived neural stem cells show lower cell proliferation	[22]
MR010	ADHD	2	Methylphenidate responder; PRS z = 1.85	9	Male		
MR014	ADHD	2	Methylphenidate responder; PRS z = 1.25	13	Male		
MR001	ADHD	2	Pediatric ADHD	15	Male *	QC only	[67]
MR010	ADHD	2	Pediatric ADHD	9	Male *		
MR013	ADHD	1	Pediatric ADHD	16	Male *		
MR014	ADHD	2	Pediatric ADHD	13	Male *		
bCJ13cl3 (UKWMPi011)	ADHD	2	Adult ADHD; SLC2A3 duplication carrier (3-copy CNV, chr12p13.31)	41	Female *	QC only	[68]
bCJ14cl1 (UKWMPi012),	ADHD	2	Adult ADHD; SLC2A3 duplication carrier (3-copy CNV, chr12p13.31)	54	Male *		
MICCNi002	ADHD	2	DSM-IV diagnosed; severe impulsivity/hyperactivity; on Ritalin	16	Male *	QC only	[69]
UKWi001-A	ADHD	1	SLC2A3 duplication carrier (12p13.31, CNV)	51	Female	QC only	[70]
MR001	ADHD	2	-	8–16	Male *	Successful reprogramming and differentiation into neural stem cells	[71]
ADHD-4	ADHD	1	DSM-5 diagnosed	-	Male	Pluripotency validated; trilineage differentiation confirmed	[72]
ADHD-5	ADHD	1	DSM-5 diagnosed	-	Male		
ADHD-10	ADHD	1	DSM-5 diagnosed	-	Male		
Deletion career 1	ADHD	3	PARK2 CNV deletion career 1	43	Female	Altered expression in pathways modulating excitation/inhibitions, oxidative stress, and related neurobiological processes	[73]
Duplication career 1	ADHD	3	PARK2 CNV duplication career 1	28	Female		
Deletion career 3	ADHD	3	PARK2 CNV deletion career 3	47	Male		
Wildtype ADHD	ADHD	3	Wildtype ADHD	30	Female		

ADHD-I—attention deficit hyperactivity disorder, predominantly inattentive type, ADHD-C—attention deficit hyperactivity disorder, combined type, PRS—polygenic risk score, CNV—copy number variation, DSM-IV/DSM-IV-TR/DSM-5—Diagnostic and Statistical Manual of Mental Disorders, Fourth Edition/Fourth Edition, Text Revision/Fifth Edition, * = Caucasian.

## Data Availability

No new data were created or analysed in this study.
